# Integrating deep learning, biological hierarchies, and high-resolution imagery to create a new identification tool for cryptic coral reef fishes

**DOI:** 10.1371/journal.pone.0349646

**Published:** 2026-06-04

**Authors:** Leonardo F. Reginato, Simon J. Brandl

**Affiliations:** Department of Marine Science, The University of Texas at Austin, Marine Science Institute, Port Aransas, Texas, United States of America; Central Marine Fisheries Research Institute, INDIA

## Abstract

Life on Earth has evolved into a staggering diversity of species, most of which still remain undiscovered, unrecognized, or unmonitored. As our ocean’s richest biodiversity hotspot, coral reefs harbor more than one third of marine biodiversity, but many reef species are small and cryptic and, therefore, difficult to identify and study. Among these, tiny bottom-dwelling (‘cryptobenthic’) fishes have been highlighted as a highly diverse (>3,000 species), understudied, and ecologically important group. However, the classification and monitoring of these fishes depend almost exclusively on the knowledge of few expert scientists, which has resulted in limited knowledge concerning the taxonomy, distribution, and population trends of these fishes. Deep learning-driven image classification—known for its ability to learn complex patterns in visual data—is an ideal candidate for automating taxonomic image classification and therefore broaden participation in ecological monitoring and biodiversity science. We developed *CryptoVision*, a new taxonomy-aware convolutional neural network with three output heads that explicitly considers taxonomic hierarchies (family, genus, species) and their biological constraints. Built on ResNet50v2 and enhanced with Squeeze-and-Excitation modules, *CryptoVision* employs a custom taxonomy-focal cross-entropy loss and four hierarchical fusion strategies (standard, concatenation, gating, attention) to assess the algorithm’s performance. Trained on a unique dataset of ~7,600 laboratory-standard and ~18,800 web-sourced images covering 113 species of small reef fishes, our tool highlights the power of integrating deep learning with innovative, taxonomically-informed design and high-resolution imagery. Indeed, *CryptoVision* achieved a ~ 25% improvement across all metrics when lab-standard imagery was incorporated and among the fusion variants, the gating approach delivered the best calibration (expected calibration error ≈ 0.01) and 90.5% average precision. Finally, guided saliency map analyses of species in the dwarfgoby genus *Eviota* illustrate that model attention can align with expert-defined morphological traits that represent critical features for species delimitation. Our results demonstrate that taxonomy-aware, multi-output deep learning on curated imagery provides a robust, interpretable framework for scalable biodiversity monitoring, ecological research, and streamlined taxonomic workflows that is particularly well-suited for the many taxa that are typically understudied due to their small size, cryptic nature, or ambiguous taxonomy.

## Introduction

Artificial Intelligence (AI), and especially deep learning, has transformed a broad range of scientific and applied fields, enabling progress in tasks such as image recognition, natural language processing, speech recognition, and the interpretation of complex biomedical data [1,2]. Building on this transformative potential, deep learning approaches have increasingly been adopted in ecology and conservation; for example, computer vision methods now automate species identification, habitat mapping, and real-time biodiversity monitoring in both terrestrial and aquatic ecosystems [3,4]. Central to these computer vision advances are convolutional neural networks (CNNs), which emerged as the foundational architecture for image analysis after AlexNet’s landmark success in achieving unprecedented classification accuracy on the ImageNet benchmark, a large-scale dataset encompassing a highly diverse set of categories across many real-world visual contexts, including animals, vehicles, people, and general objects [5]. Unlike traditional deep neural networks, CNNs employ small learnable filters—called convolutional kernels—that slide across the input, efficiently detecting local patterns and spatial features throughout the data. Stacking multiple layers of these shared kernels enables CNNs to build an abstract representation that allows for robust pattern recognition in grid-structured data, like images [1, 2, 6, 7]. The development of deeper and more efficient architectures, such as ResNet [8] and EfficientNet [9], has further increased the power and generalizability of CNNs. Notably, while CNNs were originally designed for visual tasks, their principles have proven highly effective in diverse domains, including the analysis of sound spectrograms, time series, and genomic sequences [10–12]. Despite the development of more and more advanced deep learning models and architectures, much remains to be explored regarding their capabilities, limitations, and adaptability, particularly as their use in scientific research and real-world scenarios becomes more and more accessible from a computational perspective.

AI-based tools have been extensively developed and applied in terrestrial ecosystems, where they support a wide range of biodiversity-science related tasks such as plant identification, frog acoustic classification, bird detection, and insect species recognition [13–16]. The advancements of efficient image classification architectures have accelerated the adoption of these tools in marine systems as well, enabling progress in automated identification of marine taxa. However, marine biodiversity presents a unique set of visual and logistical challenges. Underwater imaging conditions are often subject to poor lighting, turbidity, and distortion, and collecting images is complicated by limited access. Furthermore, many marine animals can undergo the rapid morphological changes, both when alive in their natural habitat and between live and preserved states. This makes visual identification of marine taxa particularly challenging and, while some species, especially in clear-water environments such as coral reefs or African rift lakes, may be readily identifiable by their striking color or body shape, many lineages have diversified into long lists of morphologically similar species that can be difficult to tell apart. As such, traditional taxonomic identification relies on expert knowledge and manual observation of key morphological traits, including subtle features such as the number of fin rays and spines, pores, or scale counts in fishes, which are time-intensive and expertise-dependent characteristics to assess. Several recent AI-based advances have improved our capacity to identify fish species with little human-based expertise [17–20], but most treat taxonomic labels as independent categories, failing to account for the hierarchical nature of biological classification and resulting in biologically implausible outputs—such as species being assigned to an incorrect family. Furthermore, models are frequently trained on heterogeneous, web-sourced imagery with variable quality, cluttered backgrounds, and inconsistent orientations, which may degrade both accuracy and interpretability. Finally, standard evaluation metrics like accuracy or precision fail to capture whether predictions preserve taxonomic coherence, which arguably presents a crucial layer of biological information that AI-based methods ought to observe. These limitations underscore the need for models that explicitly incorporate biological hierarchies, leverage high-quality standardized imagery, and adopt evaluation frameworks that reflect the structure and complexity of biodiversity data.

With approximately 30,000 described species, fishes are the most diverse group of vertebrates and account for almost half of all vertebrate species alive today [21]. They populate nearly every aquatic environment on our planet and often play critical roles for ecosystem functioning (e.g., [22]) and services, including the provision of nutritious food to human societies worldwide [23]. However, fishes are also threatened by a variety of anthropogenic stressors, most importantly overexploitation, habitat loss, climate change, and other local disturbances [24,25]. As such, comprehensive monitoring of fish biodiversity is critical, which requires the swift and efficient identification of different species.

Coral reef fishes provide a particularly interesting and potentially rewarding group of organisms for the employment of AI-driven identification. By boasting a tremendous range of colors and patterns [26], reef fishes hold high aesthetic appeal for millions of people that snorkel and dive on reefs and frequently seek to photographically capture and identify individuals [27,28]. Their striking colors and patterns offer a clear path for AI-based tools to provide near-instantaneous taxonomic identities for a wide range of species. However, there are many lineages of reef fishes in which countless species are defined by nuanced differences in appearance, including subtle divergences in body shape, color, or the arrangement of stripes, spots, lines, or dots [26], which can make it difficult for laypeople, stakeholders, and scientists alike to pinpoint species identities from photographs. Given the vast financial appeal of coral reef tourism and the rapid degradation of many reefs due to anthropogenic impacts (and with it, their aesthetic appeal [29]), providing efficient tools that can help end users identify fishes from a diverse range of photographs promises to be a worthwhile endeavor.

While much research and public attention is focused on large, conspicuous reef fish species, small, bottom-dwelling (‘cryptobenthic’) fishes commonly account for half of all species and individuals on coral reefs [30]. These fishes, which comprise more than 3,000 species across 17 core families that include the Gobiidae, Blenniidae, Tripterygiidae, Apogonidae, and others [31], are characterized by small body size (usually <5 cm), strong associations with the seafloor, morphological or behavioral crypsis, fast life cycles, and extreme mortality, which makes them an important source of animal prey for larger consumers [32,33]. These traits have also contributed to rapid and extensive diversification in many cryptobenthic lineages [31,34,35], resulting in a vast number of poorly known species that are difficult to capture, photograph, and identify, and sometimes merely differ in the most subtle morphological features. For example, most of the > 100 species in each of the two goby genera *Eviota* and *Trimma* are distinguishable only by experts that have spent decades describing, diagnosing, and revising their taxonomy [36,37]. In fact, in some cases, such as the Caribbean sponge goby *Risor ruber*, genetically divergent lineages that are recognizable as different species from a molecular perspective may lack perceptible distinguishing morphological features altogether [38]. As a result, cryptobenthic fish biodiversity has remained sparsely documented across most coral reef locations and limited resources exist to facilitate their identification. In fact, most of the world’s printed photographic ID-guides for reef fishes include, at best, a rudimentary suite of cryptobenthic fish species [cf. [39–41]], making it difficult for scientists and laypeople to accurately identify species that are encountered. This clearly hampers the study of cryptobenthic fishes, but, perhaps surprisingly, they also have strong aesthetic appeal, with more and more hobby divers and photographers specializing on macro-photography of these highly abundant, but frequently elusive species [42]. As such, there is multifaceted appeal for the development of an AI-based tool that can assist with the identification of cryptobenthic fishes and highlight the morphological traits that underpin the algorithm’s decisions: for scientists, it opens opportunities to not only illuminate the biodiversity, distribution, and biogeography of cryptobenthic fishes, but also pinpoint morphological divergences among species that may represent derived or characteristic features. For laypeople or untrained stakeholders, it bolsters the ability to identify species from photographs, which may in turn further inform scientists and conservationists regarding the biodiversity of these fishes worldwide.

To explore this opportunity, we developed *CryptoVision*, a deep learning framework designed specifically for the automated identification of a diverse variety of small-bodied, strongly reef-associated species, especially cryptobenthic fishes. Our model leverages a unique, high-quality image dataset, compiled from standardized laboratory-based photographs of freshly collected specimens alongside publicly accessible *in situ* underwater images (sourced from iNaturalist, the Smithsonian Tropical Research Institute, and FishBase) to maximize taxonomic classification accuracy and generalization. The model introduces a multi-output classification approach in which different taxonomic levels—family, genus, and species—are predicted independently and then integrated into the learning process. Furthermore, we use saliency maps to identify the morphological features that underpin the model’s decisions, and, using the goby genus *Eviota* as a model system, compare these outcomes with the diagnostic features identified in the dichotomous key to the genus [43]. In doing so, we demonstrate that *CryptoVision* contributes not only as a classification tool but also holds promise as a framework for understanding and validating machine learning predictions in the context of phylogenetically informed taxonomy and systematics.

## Methods

### Dataset preparation

We used an extensive library of standardized, laboratory-based photographs of small, bottom-dwelling (‘cryptobenthic’) fish species across several ocean basins. Each photo was obtained from specimens that were collected in the field [32,33,44,45], immediately placed on ice and then transported to the laboratory. There, each fish was placed in a small photo-tank [46] and photographed laterally against either a black or white background, facing to the left with the fins elevated whenever possible. From this digital archive, we arbitrarily pre-selected 113 target species by applying two complementary criteria. First, we required at least 30 distinct images per species to guarantee sufficient sample size for future steps. Second, we selected species to capture as much of the morphological and phylogenetic diversity inherent in our full imagery collection. Across the selected images, only adult individuals were selected; juvenile or larval stage were not included due to variation in coloration and body shape. All candidate images were organized into a standard folder hierarchy named for the corresponding family, genus, and species. These images were then subjected to our quality-control pipeline (see below), resulting in a laboratory image dataset that comprised 7,626 high-resolution images, with an average of over 67 photos per species.

To guarantee the model’s exposure to photos obtained in underwater conditions and validate its performance, we increased our dataset with web-sourced images from three trusted repositories. These web-sourced images represent real-world, uncontrolled conditions, including wide variation in lighting, pose, background complexity, and image quality. For iNaturalist (non-commercial API access), we mined every available photo for our 113 selected species using a custom script. For FishBase and the STRI database [47], we manually scraped each species page to download all displayed images. All files were then again passed through our unified quality-control pipeline. This process yielded approximately 18,800 web-based photos, approximately 71% of our total collection.

All collected images were then subjected to a quality-control pipeline to ensure consistency and to prevent the same or similar images from appearing in both training and evaluation sets. First, we computed perceptual hashes to detect and remove exact and near-duplicate frames. Next, we used automated blur (via Laplacian variance computed with OpenCV [48]) and size checks to flag outlier images that were below our sharpness or minimum-resolution threshold for manual review and, if appropriate, removal. Every image’s label was cross-checked against its visible diagnostic traits (fish shape, body proportions, color patterns), discarding any label-morphology mismatch, suboptimal framing, or ambiguous anatomy. Finally, we manually applied a square crop to all web-sourced images, preserving fish natural morphology while eliminating distracting background clutter. After this multi-stage data QCQA process, our combined dataset contained approximately 26,500 images that passed checks and were standardized for further augmentation and model training.

The final *CryptoVision* dataset comprised images across 20 taxonomic families, 62 genera, and 113 species. To support robust training and unbiased evaluation, we partitioned these images into training (70%), validation (15%), and test (15%) subsets. These splits were stratified at the species level—each species retains the same relative representation in every subset as in the full dataset—ensuring that even the rarest taxa appear in all three sets and preventing “unseen” species during validation or testing.

### Model architecture

**Fig 1:**
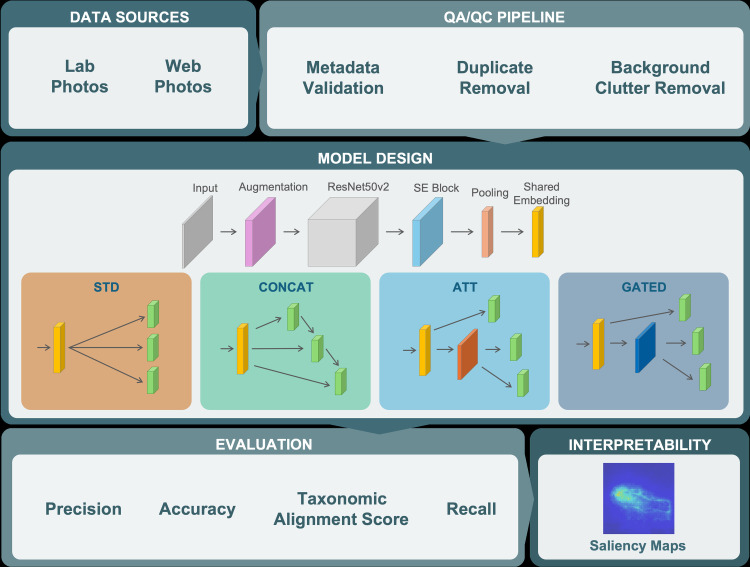
End-to-End CryptoVision Workflow. Overview of the workflow including image acquisition from laboratory and web sources, QA/QC filtering, and model training with four fusion strategies—standard (STD), feature concatenation (CONCAT), attention (ATT), and gated (GATED). Models are evaluated using precision, recall, accuracy, and our custom Taxonomic Alignment Score (TAS), and interpreted via saliency maps.

To develop *CryptoVision*, we built upon a standard convolutional model with specialized modules for robust, taxonomically-informed fish classification. We began with an ImageNet pretrained model working as our feature extractor and inserting an augmentation pipeline to improve generalization and mitigate the small number of images per class. Then, we attached three parallel “heads” to predict family, genus and species simultaneously (Fig 1). To respect the hierarchical relationships among taxonomic levels, we introduced both novel loss functions and fusion blocks that allow higher‐level predictions to inform and correct lower‐level decisions.

To maximize generalization and reduce overfitting on our relatively small lab‐standard archive and more variable web imagery, we applied several image transformations during the training stage. Specifically, following established data augmentation practices in computer vision tasks for image limited datasets [49], we implemented an augmentation block (implemented via TensorFlow-Keras) including the following settings: random horizontal flips and rotations (±10%), random zoom (height/width factors 5–10%), random contrast (±20%) and brightness (±20%), random translation (±10% in both axes), a final random crop back to 352x352 pixels, and Gaussian noise (σ = 0.2). Because these operations occur in GPU memory only during training, we avoided inflated storage requirements while still presenting the model with thousands of unique “views” of each specimen.

We selected ResNet50v2 (ImageNet-pretrained model; [8] as our feature extractor due to its high initial precision, recall and accuracy and seamless integration with gradient-based interpretability tools, and a moderate parameter size (~25M). From this pretrained model we obtained a matrix of convolutional feature maps, which we then recalibrated via a Squeeze-and-Excitation (SE) block [50], characterized by the following steps sequence: 1) Squeeze: global average pooling collapses each feature channel to its mean activation. 2) Excitation: two fully connected layers—with ReLU activation after the first layer and sigmoid activation after the second—learn per-channel weights. 3) Re-scale: these weights multiply the original feature maps, amplifying more important channels. The SE-recalibrated feature maps were then reduced to a fixed-length vector by global max-pooling. We fed this vector through a shared dense layer (2048 neurons, ReLU activation and 0.3 dropout) to produce our shared embedding. This task-agnostic embedding serves as the input to all downstream taxonomic heads (family, genus, and species).

To leverage the inherent taxonomic hierarchy in nature and extract the full potential of a single deep learning model, we designed our classifier with three parallel “heads” immediately after the shared embeddings. This so-called multi-output approach created three fully connected blocks (dense layers with SoftMax activation), which map the previously shared vector into a probability distribution over its own label set. By reusing the same embeddings across all three prediction tasks, we not only reduce the model size, but also force the model to learn representations that are simultaneously useful at coarse (family) and fine (species) granularity. This multi-task arrangement lets information flow between ranks (e.g., family cues strengthen genus predictions), removes the need for separate models at each level, and ultimately yields independent confidence scores for each taxonomic level (family, genus and species).

To enhance alignment across family, genus, and species outputs, we extended our multi-output model with tree “fusion” strategies, each connected with the same shared embedding but differing in how they inject higher-level context into downstream heads. We compared these against a standard (STD) baseline that shared no information between predictions. Specifically, we applied the following designs to compare their influence on model outputs:

- STD (Standard Multi-Output): In the simplest configuration, each head considers only the shared embedding and operates independently.
- CONCAT (Logit Concatenation): The embedding vector concatenates with the family head’s raw class scores (its “logits”). The same procedure is applied to the genus prediction, where the genus head receives the shared embedding concatenated with the family logits; similarly, the species head input combines the shared embedding with the genus logits.
- GATED (Learned Gate Fusion): Instead of directly using the SoftMax output from the family prediction as input, we added a sigmoid “gate” for each embedding dimension that dynamically balances the original features against a transformed version of the family logits. The gate is also created between genus and species layers.
- ATT (Taxonomy-Conditioned Attention): For this design, we added an “attention mask” (via a small two-layer perceptron with sigmoid activations) for which values between 0 and 1 indicate how relevant each feature dimension should be for the genus predictions. By multiplying this mask with the embedding layer, we highlight channels that are most diagnostic for the family output, a process comparable to pinpointing the features that matter most given the higher-level context. The same genus attention-mask was created and coupled to species head input.

All four designs were implemented and compared in terms of their accuracy, precision, recall, and the Taxonomic Alignment Score (TAS). By progressively training and testing all these architectures, we were able to measure how much each fusion style improves hierarchical consistency and overall classification performance, ultimately revealing the optimal design for our hierarchical classification tasks.

Given that our dataset had some imbalances (between 90–270 images per species), standard cross-entropy may bias the model towards over-represented taxa and potentially ignore the strict relationships that are inherent to biological classification (i.e., misassign a species to the wrong genus or family). To address this, we developed a custom loss function—Taxonomy-Focal Cross-Loss (TFCL)—which combines class reweighting through focal loss with soft penalties that encourage hierarchical consistency across taxonomic ranks. TFCL builds upon Focal Cross-Entropy, which augments the standard cross-entropy with a modulating factor to down-weight easy examples and focus learning on harder instances [51]:


$$\begin{array}{c} L_{focal}\left(p_{t}\right)=-\alpha {\left(\text{1}-p_{t}\right)}^{\gamma }*\;{\text{log}(p}_{t}) \end{array}$$ (1)

Here, $p_{t}$ is the model’s estimated probability for the true class, α is the balance between positive and negative examples and γ is the focusing parameter, adjustable to learn on hard misclassified cases. We extended this by adding a *soft consistency* penalty that encourages each lower-level head to “agree” with its parent heads. Specifically, we implemented it where:

- $\overset{fam}{\underset{i}{p}}$, $\overset{fam}{\underset{j}{p}}$, $\overset{fam}{\underset{j}{p}}$ are the SoftMax scores over families i, genera j, and species k
- $y_{fam},\;y_{gen},\;y_{spe},$their true labels,
- ${par}_{gen}(j)$ the parent family of genus *j*, and ${par}_{spe}(k)$ the parent genus of species *k*.

Using these penalties, the loss for a single sample is computed as:


$$\begin{array}{c} L_{fam}=\;-\alpha {\left(\text{1}-\overset{fam}{\underset{y_{fam}}{p}}\right)}^{\gamma }\text{log}\overset{fam}{\underset{y_{fam}}{p}} \end{array}$$ (2)

$$\begin{array}{c} L_{gen}=\;-\alpha {\left(\text{1}-\overset{gen}{\underset{y_{gen}}{p}}\right)}^{\gamma }\text{log}\overset{gen}{\underset{y_{gen}}{p}}+\;\lambda_{gen}[-\text{log}\overset{fam}{\underset{{par}_{gen}(y_{gen})}{p}}] \end{array}$$ (3)

$$\begin{array}{c} L_{spe}=\;-\alpha {\left(\text{1}-\overset{spe}{\underset{y_{spe}}{p}}\right)}^{\gamma }\text{log}\overset{spe}{\underset{y_{spe}}{p}}+\;\lambda_{spe}[-\text{log}\overset{gen}{\underset{{par}_{spe}(y_{spe})}{p}}] \end{array}$$ (4)

Following this, our final loss is computed as:


$$\begin{array}{c} L_{TFCL}=L_{fam}+L_{gen}+L_{spe} \end{array}$$ (5)

By combining focal reweighting with hierarchy-aware penalties, *TFCL* drives the model to excel on rare taxa and to maintain biologically valid predictions across all three taxonomic levels.

### Experimental design and evaluation

We conducted two complementary analyses to isolate the contributions of our standardized lab image dataset and the hierarchy-aware fusion designs to overall model output quality. All runs shared the same general training setup, which used ResNet50v2 connected with the SE Block for feature enhancement, followed by a global-max-pooling layer to combine the three parallel SoftMax heads (family, genus and species). All training methods also used our TFCL and shared the same hyper-parameters. We report common classification metrics (accuracy, precision, recall) at each taxonomic level, plus a newly derived Taxonomic Alignment Score (TAS), which directly measures taxonomic consistency among the hierarchical levels. Specifically, while traditional metrics (precision, recall and accuracy) capture each head’s performance in an isolated fashion, TAS quantifies whether the three predictions form a biologically valid family-genus-species chain. Concretely, a test example (i) with predicted family ($f_{i}$), genus ($g_{i}$) and species ($s_{i}$) is valid only if:


$$\begin{array}{c} parent\left(g_{i}\right)=f_{i}\;\wedge \;parent\left(s_{i}\right)=g_{i} \end{array}$$ (6)

We define TAS as the fraction of samples satisfying both conditions, as detailed by:


$$\begin{array}{c} TAS\frac{\text{1}}{N}\sum_{i=\text{1}}^{N}\text{1}[parent\left(g_{i}\right)=f_{i}\wedge parent\left(s_{i}\right)=g_{i}], \end{array}$$ (7)

Where the parent comparison is the indicator (1 if true, 0 otherwise). A perfect TAS = 1 means every genus falls within its predicted family and every species within its predicted genus. In turn, lower values indicate cross-level inconsistencies that violate biological relationships.

To specifically test the effect of our proprietary lab-based image archive, we trained two classification models using two size-matched datasets of either web only images (100% web-source images) or a 50:50 mixed composition (half of the images from the web, while the other half was from our laboratory-based images). We limited this experiment to species that had at least 100 web-sourced and 50 lab-based images. Thus, for the web-only run, all species had 100 images, while in the mixed set, we randomly replaced 50 web-based images with 50 lab-based images, while retaining the same number of images per species, total dataset sizes, and a balanced number of images for each run.

We next evaluated the impact of hierarchy-aware fusion, by comparing our four model variants under identical training conditions. All experiments used the full *CryptoVision* dataset, split into 70% training, 15% validation, and 15% test. Aside from the fusion strategy, all hyperparameters were identical (ResNet50v2 with SE block, input resolution (352x352 pixels), dropout = 0.3, batch size = 32), and all random seeds were set to be the same. Each model had two training stages, consisting of 1) 15 training epochs with all pretrained layers frozen, updating only the shared embedding and taxonomic heads, and 2) a fine-tuning stage in which the top 75 pretrained layers were unfrozen and trained with an additional ten epochs, allowing high-level features to adapt while preserving low-level stability. By keeping all other parameters constant, this protocol ensures that any differences in classification metrics and the TAS arise solely from the choice of STD, CONCAT, GATED, or ATT fusion.

To interpret the morphological features driving the model’s predictions and compare their alignment with expert-defined traits, we generated saliency maps—visual explanations that indicate which regions of an input image most influence the model’s output. These maps are computed by taking the gradient of a given model output with respect to each input pixel [52,53]. Thus, this method measures how each pixel affects the prediction’s confidence, with pixels with higher gradient magnitudes representing those that the model relies on most heavily to make its decision. To enhance interpretability via suppression of noise from irrelevant gradients, we applied guided backpropagation [54,55], which restricts the saliency visualization to positive contributory activations in the network. Saliency computation was implemented using the tf-keras-vis library [56], with a linearization step to ensure compatibility with our model and explicitly targeting the species output head.

Finally, we assessed whether model attention aligns with expert-defined diagnostic traits. To do so, we focused our saliency analysis on a subset of correctly classified test images from the dwarfgoby genus *Eviota*, the most abundant and taxonomically diverse group in our dataset. Specifically, *Eviota* now contains more than 130 species, which are unified by similar anatomical and morphological features but often differ in subtle aspects of their coloration or patterning [35,57]. Using diagnostic descriptions from [36]), which represents the most comprehensive and detailed dichotomous key for the genus, we compared the high-saliency regions identified by the model with externally visible, image-based diagnostic traits used in species identification, such as coloration patterns, pigment markings, body regions, and anatomically salient body features (e.g., head, eye, and caudal regions). The resulting saliency maps were normalized and overlaid on the original images to facilitate visual inspection and comparison.

## Results

### High-quality image effect

**Fig 2:**
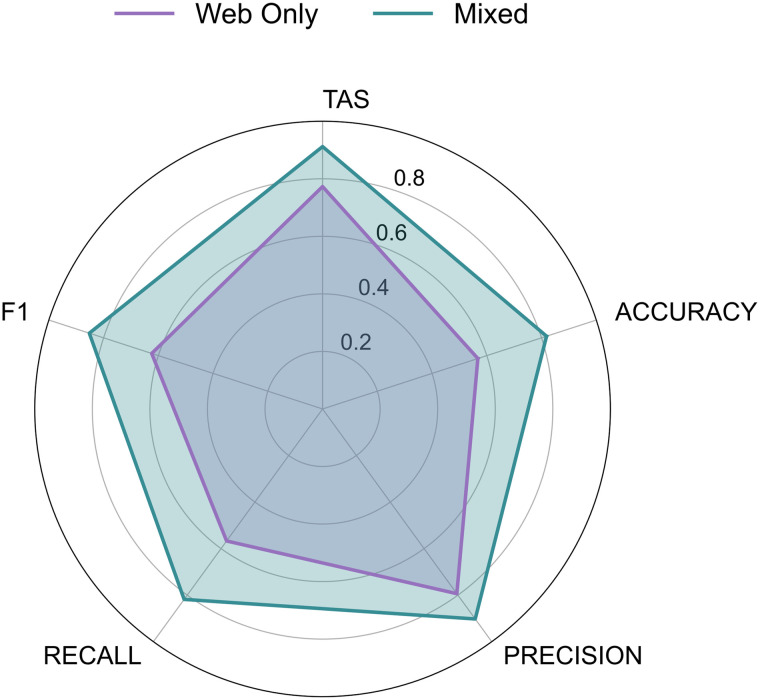
Model performance comparison across data sources. Radar plot comparing models trained on Web Only (purple) vs. Mixed (green) datasets. Vertices reflect the different metrics, all of which are constrained between 0 and 1. TAS = Taxonomic Alignment Score; F1 = Harmonic mean between precision and recall.

We compared models trained on two datasets—Web-Only and Mixed (web plus high-quality lab images)—to evaluate how image quality influences overall model performance. The Mixed model consistently outperformed the Web Only model across all metrics (Fig 2). The most pronounced improvement was observed in both Recall and Accuracy (which increased by approximately 25%), indicating that the inclusion of high-quality images improves the model’s sensitivity to minority or difficult classes, reducing false negatives. Additionally, the TAS increased by 14%, reflecting better consistency across hierarchical predictions.

**Fig 3:**
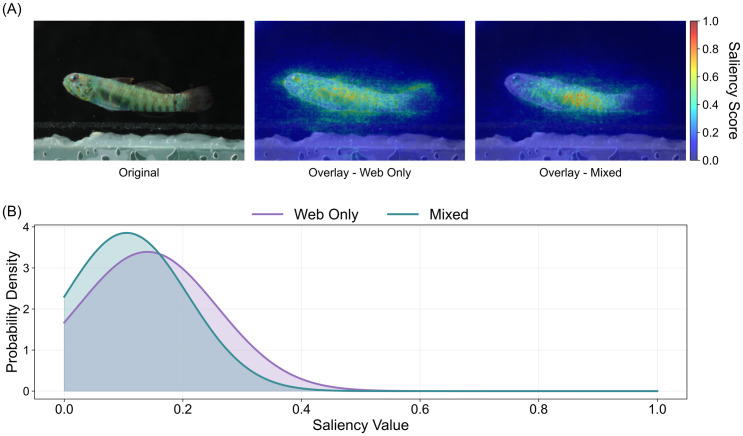
Training dataset influences model attention patterns and saliency distributions. **(A)** Saliency map overlays for a cryptobenthic fish image of the dwarfgoby Eviota afelei. Photos (left to right) show the original image, the saliency map overlay from a model trained on only web-based images, and the overlay from the model trained with the lab-based images. Colors represent the saliency score. Original photograph taken by the authors as part of the laboratory-standard image dataset used in this study. **(B)** Distribution of saliency values from the maps displayed in the image.

Furthermore, using qualitative saliency visualizations, we examined how the model’s attention is spatially distributed across the fish body when trained on the two different datasets (Fig 3). The Web-Only saliency map overlay exhibited a perceptibly noisier and more dispersed signal, activating both relevant anatomical regions and background artifacts. In contrast, the Mixed model showed more spatially constrained and localized attention on the fish-body patterns (Fig 3a).

These visual trends were quantitatively supported by the saliency‐value distributions computed across the images sample (Fig 3b). The Mixed model exhibited a more concentrated profile, with a sharply peaked distribution (mean ≈ 0.099; standard deviation ≈ 0.100), indicating highly focused attention on a small subset of pixels. By contrast, the Web-Only model’s distribution was noticeably broader (mean ≈ 0.129; standard deviation ≈ 0.108), indicating a more diffuse and less discriminative allocation of saliency.

### Hierarchical architecture comparison

**Fig 4:**
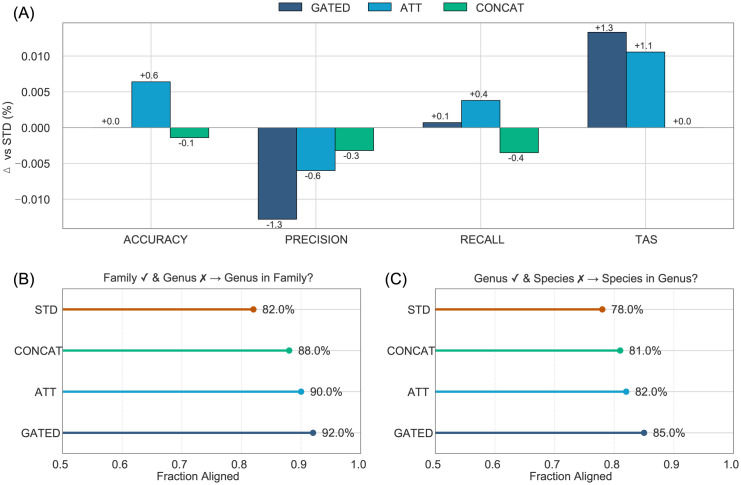
Relative model performance and taxonomic consistency. **(A)** Model performance relative to the standard (STD) baseline (Δ) across the core metrics—Accuracy, Precision, Recall—and the Taxonomic Assignment Score (TAS). **(B)** Fraction of genus-level misclassifications that still map to the correct family. **(C)** Fraction of species-level misclassifications that still map to the correct genus.

To assess the value of our hierarchical model architecture, we first focused on the overall metrics between the standard and all three fusion schemes (based on concatenated family–genus–species predictions). While all models achieved very similar overall accuracy and recall, the baseline STD scored the highest precision, and both GATED and ATT substantially outperformed the other schemes in TAS (Table 1). Delta scores of the different metrics compared to the baseline (STD) highlighted a possible trade-off between improving TAS and decreasing the overall precision (Fig 4a). While marginally so, GATED and ATT also exceeded STD in accuracy, recall, and TAS, three out of four available metrics.

**Table 1:** Mean metrics of different model architectures when run on the test set.

Models	Precision	Recall	Accuracy	TAS
**STD**	0.9182	0.8625	0.8321	0.9414
**CONCAT**	0.9150	0.8590	0.8307	0.9414
**GATED**	0.9054	0.8632	0.8321	0.9547
**ATT**	0.9122	0.8663	0.8385	0.9519

We then examined the hierarchical awareness for links between family-genus and genus-species by asking how frequently the model assigns misclassifications at the lowest taxonomic level to the correct higher taxonomic rank (i.e., how often is an incorrectly classified species assigned to the right genus)? GATED improved this metric substantially, exhibiting an increase of up to 10% compared with other methods, demonstrating that GATED is far more likely to suggest a closely related species even when it misses the exact label. In turn, STD showed by far the lowest score, highlighting the utility of any fusion strategy for hierarchical classification (Fig 4b and 4c).

To determine whether the observed differences in overall accuracy between fusion strategies reflect systematic performance improvements, we conducted paired McNemar’s tests on the test set. No statistically significant differences were detected between the GATED architecture and any alternative fusion scheme (STD, CONCAT, ATT; all p ≥ 0.12), despite small numerical differences in accuracy (ΔAcc ranging from −3.7% to +0.5%). In all comparisons, disagreement counts were approximately symmetric, indicating similar error patterns rather than consistent gains in exact classification performance.

**Fig 5:**
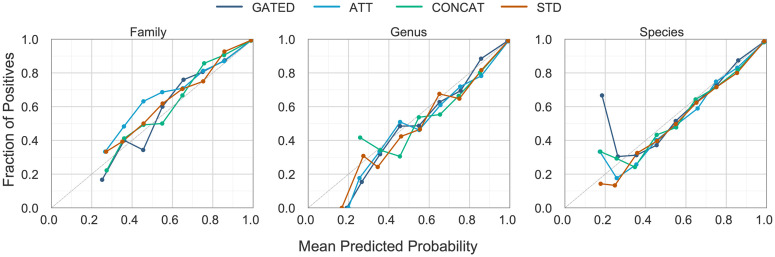
Calibration of model predictions across taxonomic tasks. Reliability diagrams for the three taxonomic prediction tasks, showing the empirical fraction of positives (y-axis) versus the mean predicted probability (x-axis). Each colored curve corresponds to one model variant—GATED (dark blue), ATT (cyan), CONCAT (green) and STD (orange)—while the dashed diagonal line marks perfect calibration.

Finally, we assessed model calibration via reliability diagrams (Fig 5) alongside the expected calibration error (ECE). A well-calibrated model’s predicted confidence should align with its observed accuracy, and ECE quantifies the average gap between them. Although all four models exhibit generally low calibration errors, GATED delivered the strongest performance (ECE = 0.0108 (family), 0.0084 (genus), and 0.0107 (species), averaging ≈ 0.01). In contrast, ATT showed the highest errors (0.0124, 0.0133, and 0.0151 at family, genus, and species, respectively (average ≈ 0.0135)), suggesting a tendency toward overconfidence despite its strong TAS performance.

Taken together, these analyses show that the GATED fusion strategy delivers the best overall balance, providing substantially higher cross-level consistency (TAS) and genus-to-species alignment, at only a minor precision cost, and with the best calibration, thus making it the preferred design for taxonomy-aware deep classification.

To investigate model performance beyond aggregate accuracy metrics, we conducted a per-species error analysis based on recall-derived error rates (1 − recall). Species were grouped into three categories according to their classification error: low (0–10%), moderate (10–25%), and high (>25%) error classes (S1–S3 Figs). The majority of species (58 of 113) fell into the low-error category, exhibiting consistently high recall with error rates below 10%. An additional 36 species showed moderate error rates, while 19 species exhibited substantially reduced performance, with error rates exceeding 25%.

Species in the high-error category showed persistent difficulty in being distinguished based on visual features alone, often coinciding with strong visual similarity among closely related taxa. To assess whether taxonomic complexity was associated with classification performance, we evaluated the relationship between species-level error and genus richness (number of species per genus; S4 Fig.). A weak positive trend was observed, with error rates tending to increase with genus richness; however, this relationship was modest (Pearson’s *r* = 0.228). Together, these results indicate that, while more diverse genera are more challenging to classify, per-species classification performance is not explained by genus richness alone.

### Saliency maps & trait overlaps

Comparing and contrasting saliency maps with morphological features outlined in taxonomic keys revealed broad overlap, with model attention frequently highlighting even subtle diagnostic features. Below, we display three examples of *Eviota* species that exemplify this alignment.

**Fig 6:**
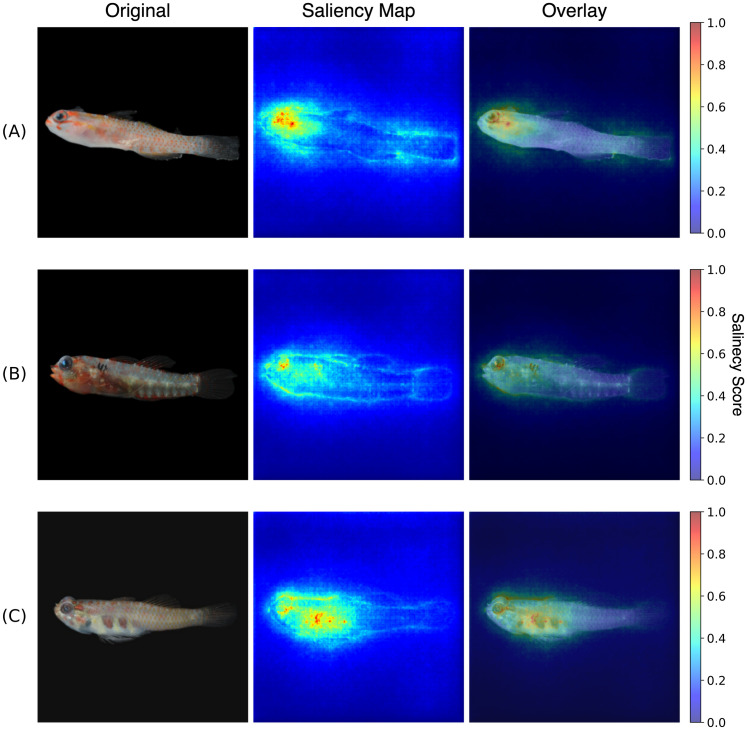
Representative saliency-map outputs for selected species. Saliency map for three fish specimens: **(A)** Eviota albolineata, **(B)** Eviota infulata, and **(C)** Eviota teresae. In each row, panels show (left) the original input image, (middle) the normalized saliency map (pixel importance scores from 0.0–1.0), and (right) the saliency heatmap overlaid on the original image. Original photographs taken by the authors as part of the laboratory-standard image dataset used in this study.

A clear example of trait alignment is observed in the saliency map of the whitelined dwarfgoby *Eviota albolineata* (Fig 6a), where model attention is concentrated around the head, eye, and upper pectoral-fin base. These focal areas coincide precisely with the species’ main diagnostic characters as described by [36], which state: *“Two unbroken stripes behind eye, upper across nape, lower across operculum”* and *“oblique wide stripe of melanophores across center of pectoral-fin base”.* The model’s focused attention on these regions indicates that it has learned to prioritize the same visual traits that taxonomists use to distinguish this species.

In *Eviota infulata* (Fig 6b), the saliency map highlights the upper anterior body, just above the pectoral-fin base. This region corresponds precisely to the highly characteristic W-shaped black mark that is primarily used to identify the species. As described by [36], diagnostic characters include: “*Irregular or W-shaped black mark on upper anterior body above and just posterior to base of pectoral fin”*, *“no distinct black spot at caudal-fin base”*, and *“7 postanal ventral-midline dark spots from subcutaneous body bars”.* While the model’s saliency map shows minimal activation on the caudal fin—consistent with the absence of a defining mark—moderate saliency in other body areas suggests that broader morphological context is also considered. This balance between localized and distributed attention reinforces the model’s interpretability and its nuanced approach to species classification.

Finally, in *Eviota teresae* (Fig 6c), the saliency map is strongly focused on the abdomen and upper part of the eye, which align well with the species’ diagnostic traits. As described by [36], these include: *“reddish blotches on abdomen taller than wide”*, *“dorsal part of eye reddish with small spots”*, and *“No prominent dark spots on body along base of dorsal fins”*. The reduced saliency along the dorsal midline mirrors the absence of defining features in this body region, reinforcing the model’s sensitivity not only to prominent traits but also to their absence. This example highlights how even fine-scale pigment patterns are integrated into the GATED model’s classification strategy, illustrating its capacity to combine expert-level trait recognition with broader pattern synthesis.

## Discussion

The development of *CryptoVision* represents a significant step in the use of biologically-informed deep learning for taxonomically structured image classification in marine biodiversity. By combining a multi-output CNN architecture with a custom loss function and biologically-informed and interpretable tools, our framework not only achieves strong performance across hierarchical taxonomic levels but also offers unique insights into the saliency of morphological features in species classification. Our results reveal the potential and limitations of applying such models in highly diverse taxonomic groups, such as cryptobenthic coral reef fish lineages, in which accurate identification of closely related species cannot be achieved by laypeople due to the lack of reference material and the need for taxonomic expertise. Finally, our results unlock potential applications of deep learning for the interpretation of synapomorphies or subtle color pattern differences in biodiversity science, as refined, highly trained models may in fact complement human recognition for the identification of key morphological features and thus aid taxonomists in their work.

The development and implementation of the Taxonomic Alignment Score (TAS) offers a novel evaluation metric that quantifies whether predictions form biologically coherent taxonomic chains across family, genus, and species. While traditional metrics such as accuracy, precision and recall are widely used in classification models [13,15,16,58–60], they evaluate each label independently and fail to capture cross-level taxonomic consistency. TAS addresses this gap by evaluating hierarchical alignment, making it especially valuable in ecological and phylogenetic applications where misclassification across taxonomic levels can distort biological interpretation. When combined with our Taxonomy-Focal Cross-Loss (TFCL)–which enforces cross-level agreement during training–TAS enables both evaluation and optimization to be grounded in biologically reasonable structures, while also helping to mitigate the effects of class imbalance by leveraging shared information across taxonomic levels. As multi-output architectures and hierarchically oriented models gain traction in ecological classification [14,15,58], metrics like TAS will become important to ensure ecological and evolutionary relevance.

Our findings also underscore the importance of standardized, high-quality imagery. The mixed dataset (combining lab-standard and web images) led to substantial performance gains—averaging nearly 30% improvement across all core metrics—when compared to the model trained on web-only images. This result aligns with prior studies on the impact of image quality in computer vision [61–63], and confirms that high-resolution, consistently oriented images enhance not only classification accuracy but also model interpretability, especially for complex systems such as biodiversity images. As demonstrated by the saliency maps, the mixed model that included lab-based images revealed more selective attention distribution, suggesting that clean training imagery enables the model to extract finer-scale features relevant for taxonomic decisions. Thus, the use of high-resolution, standardized photographs with little noise greatly enhances model trustworthiness, which is essential if advances in deep learning techniques are to become more widely implemented in scientific research.

Although hierarchical fusion designs like GATED and ATT resulted in modest improvements (~1%) in conventional performance metrics, these differences were not statistically significant under paired McNemar testing (p ≥ 0.12). Nevertheless, both approaches provided notable gains in taxonomic alignment and cross-level consistency. This outcome contrasts with prior studies in fishes and other taxa such as frogs and parrots [14–16,58], where hierarchical fusion approaches led to more substantial improvements across traditional metrics, including precision and accuracy. Direct comparisons, however, must be interpreted with caution, as differences in model architecture (e.g., network depth or type of fusion mechanism), dataset size and quality, and the inherent diversity and complexity of the taxonomic groups involved can all influence the effectiveness of hierarchical designs. In particular, cryptobenthic fishes present a challenging classification target due to subtle morphological differences and frequent trait overlap among species, which may limit the extent to which hierarchical learning translates into gains in raw classification performance. This interpretation is supported by the per-species error analysis, which revealed substantial heterogeneity in classification performance across taxa, with higher error rates concentrated among visually similar and closely related species. These results highlight that aggregate accuracy metrics can obscure class-level difficulty in fine-grained taxonomic classification.

Nonetheless, our results demonstrate that incorporating hierarchical design principles yields clear benefits when evaluated through alignment-focused metrics. While TAS improved by approximately 1.3%, the most pronounced gains were observed in family-to-genus and genus-to-species alignment scores, which increased by an average of 10% compared to the baseline STD model. These improvements highlight the value of hierarchical fusion in promoting biologically coherent predictions, even when effects on conventional accuracy and precision are modest. Notably, despite introducing only a slight increase in model parameters (~0.03%), GATED and ATT architectures maintained computational efficiency, underscoring that even small architectural modifications can yield biologically meaningful improvements.

Our saliency map analysis further provides insights into the model’s internal process, displaying consistent overlap between attention regions and morphological traits documented by expert taxonomists [36]. Given the absence of standardized quantitative metrics for evaluating saliency correctness with fine-grained taxonomic classification, we used the genus *Eviota* as a representative case study, to qualitatively assess alignment between model attention and expert-defined diagnostic traits. Using this case study, the GATED model demonstrated a clear focus on species-specific features such as the W-shaped shoulder mark in *E. infulata* and opercular striping in *E. albolineata*. Moreover, the model’s attention varied across species, indicating that it had learned to recognize and use different traits depending on the input image. Broader features such as body shape and fin structure, were also highlighted, suggesting a hybrid strategy that integrates both localized diagnostic cues and generalized visual context. This mirrors human taxonomic reasoning and illustrates how explainability tools like saliency maps can help to illustrate the decision-making processes of deep learning models. As we continue to explore and investigate the hidden biodiversity of our oceans, developing and improving tools to aid with the identification of salient morphological features promises to be a useful endeavor for scientists.

In summary, our study demonstrates that taxonomy-aware deep learning models, when coupled with hierarchical loss functions, quality-controlled image datasets, and interpretable outputs such as saliency maps, can serve as powerful tools for marine species classification. Indeed, although the perception mechanisms of humans and deep learning models are fundamentally different, the pattern of attention exhibited by *CryptoVision*—guided by both broad morphological characteristics and species-specific, externally visible diagnostic traits—show remarkable alignment with expert taxonomists, suggesting great scope in the use of models such as the one developed herein for the general public, stakeholders, and scientists. While challenges remain–including inherent class imbalance, annotation consistency, and limited availability of high-quality images for rare taxa–the integration of performance, interpretability, biological alignment, suggest this as a promising path for implementing AI in biodiversity research. As ecological monitoring becomes increasingly automated and our needs to understand and monitor biodiversity outpace the number of scientists with sufficient expertise, tools like *CryptoVision* may play a useful role in scaling taxonomic identification and advancing our understanding of cryptic biodiversity in marine ecosystems.

## Supporting information

S1 Fig Per-species classification error in the low-error category (0–10%). Species-level classification error (1 – recall) for species with error rates between 0 and 10%. Each point represents one species, ordered by increasing error. The y-axis shows classification error (1 – recall), and the x-axis lists species names. A total of 58 species fall within this category.
(TIFF)

S2 Fig Per-species classification error in the moderate-error category (10–25%). Species-level classification error (1 – recall) for species with error rates between 10% and 25%. Each point represents one species, ordered by increasing error. The y-axis shows classification error (1 – recall), and the x-axis lists species names. A total of 36 species fall within this category.
(TIFF)

S3 Fig Per-species classification error in the high-error category (>25%). Species-level classification error (1 – recall) for species with error rates greater than 25%. Each point represents one species, ordered by increasing error. The y-axis shows classification error (1 – recall), and the x-axis lists species names. A total of 19 species fall within this category.
(TIFF)

S4 Fig Relationship between species-level classification error and genus richness. Species-level classification error (1 – recall) plotted against genus richness (number of species within each genus). Each point represents a single species. The dashed red line indicates the linear regression trend (slope = 0.0111), and Pearson’s correlation coefficient is r = 0.228.
(TIFF)
