# Supplementary figures and images for: Integrating deep learning, biological hierarchies, and high-resolution imagery to create a new identification tool for cryptic coral reef fishes
Source: PLoS One. 2026 Jun 4;21(6):e0349646. doi: 10.1371/journal.pone.0349646 (PMC13235906; doi:10.1371/journal.pone.0349646)

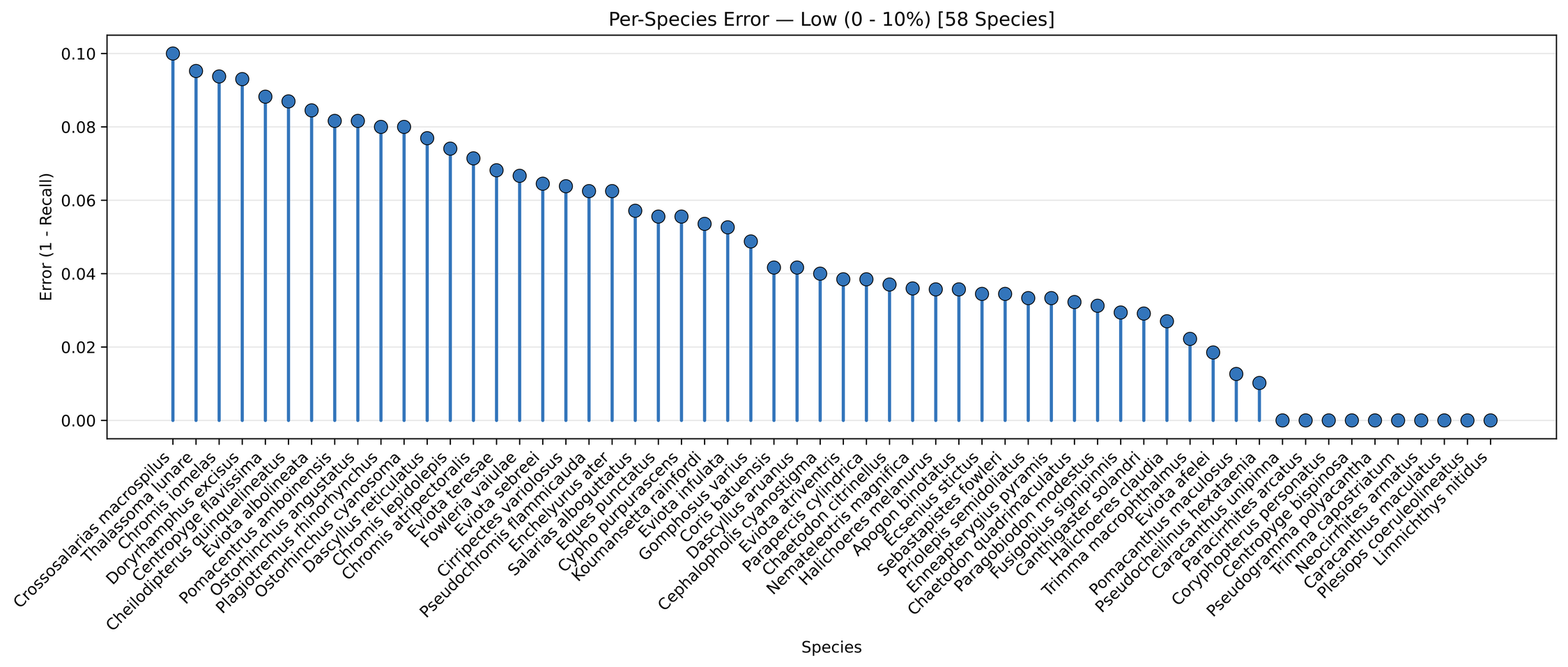

Supplement: S1 Fig — Species-level classification error (1 – recall) for species with error rates between 0 and 10%. Each point represents one species, ordered by increasing error. The y-axis shows classification error (1 – recall), and the x-axis lists species names. A total of 58 species fall within this category. (TIFF) [file pone.0349646.s001.tiff]

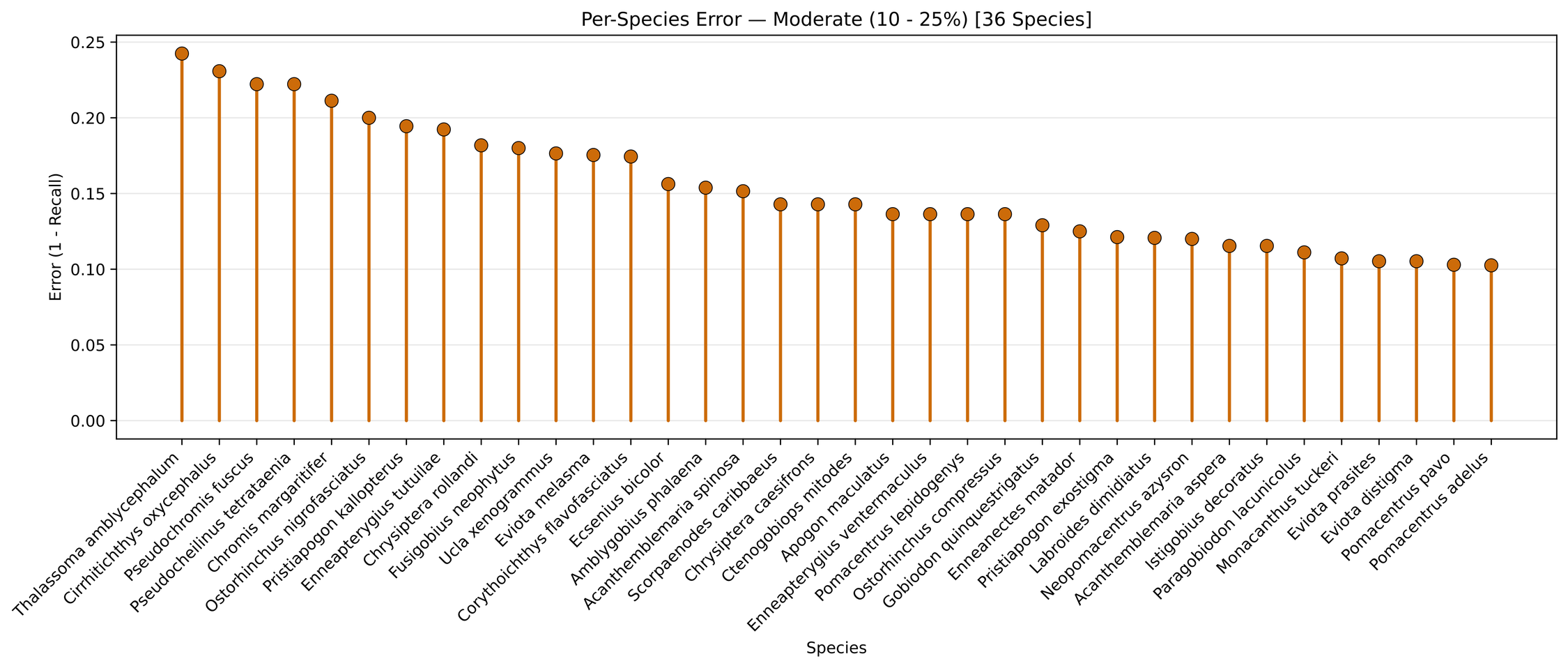

Supplement: S2 Fig — Species-level classification error (1 – recall) for species with error rates between 10% and 25%. Each point represents one species, ordered by increasing error. The y-axis shows classification error (1 – recall), and the x-axis lists species names. A total of 36 species fall within this category. (TIFF) [file pone.0349646.s002.tiff]

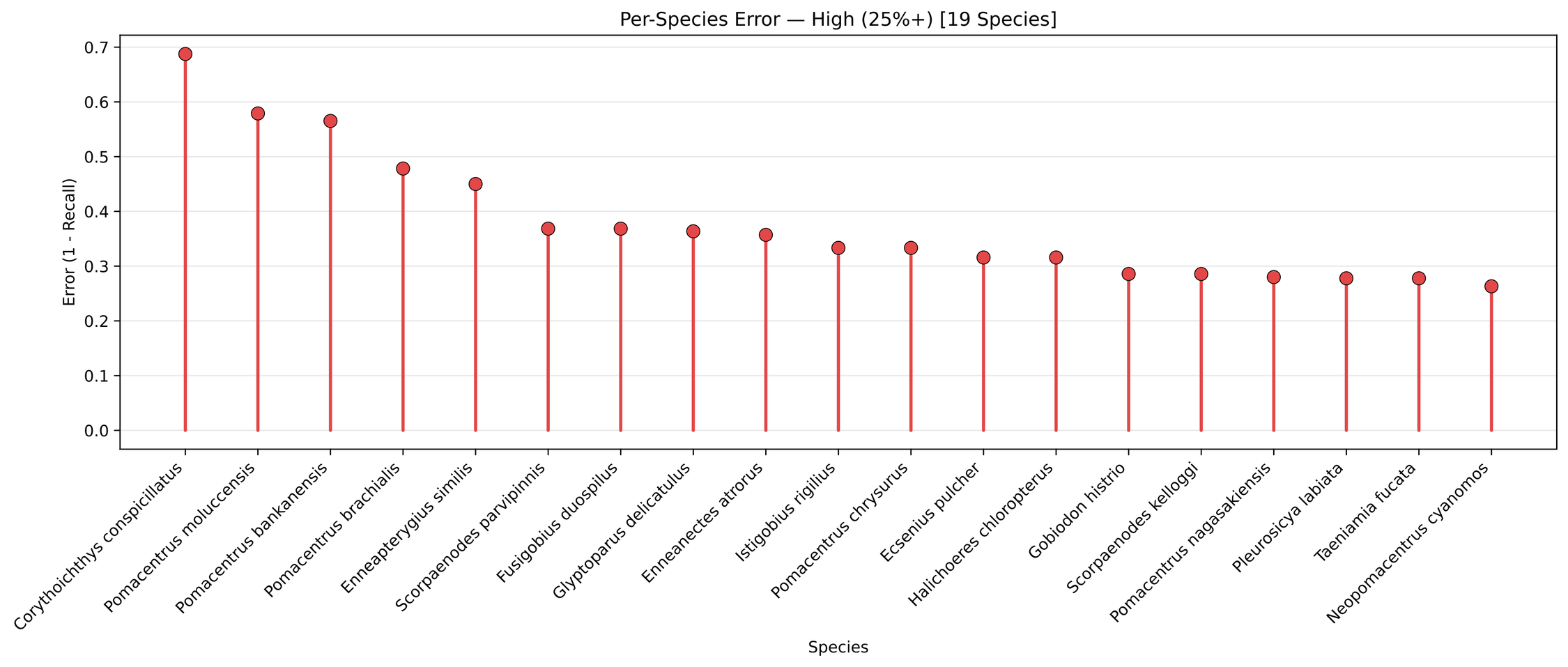

Supplement: S3 Fig — Species-level classification error (1 – recall) for species with error rates greater than 25%. Each point represents one species, ordered by increasing error. The y-axis shows classification error (1 – recall), and the x-axis lists species names. A total of 19 species fall within this category. (TIFF) [file pone.0349646.s003.tiff]

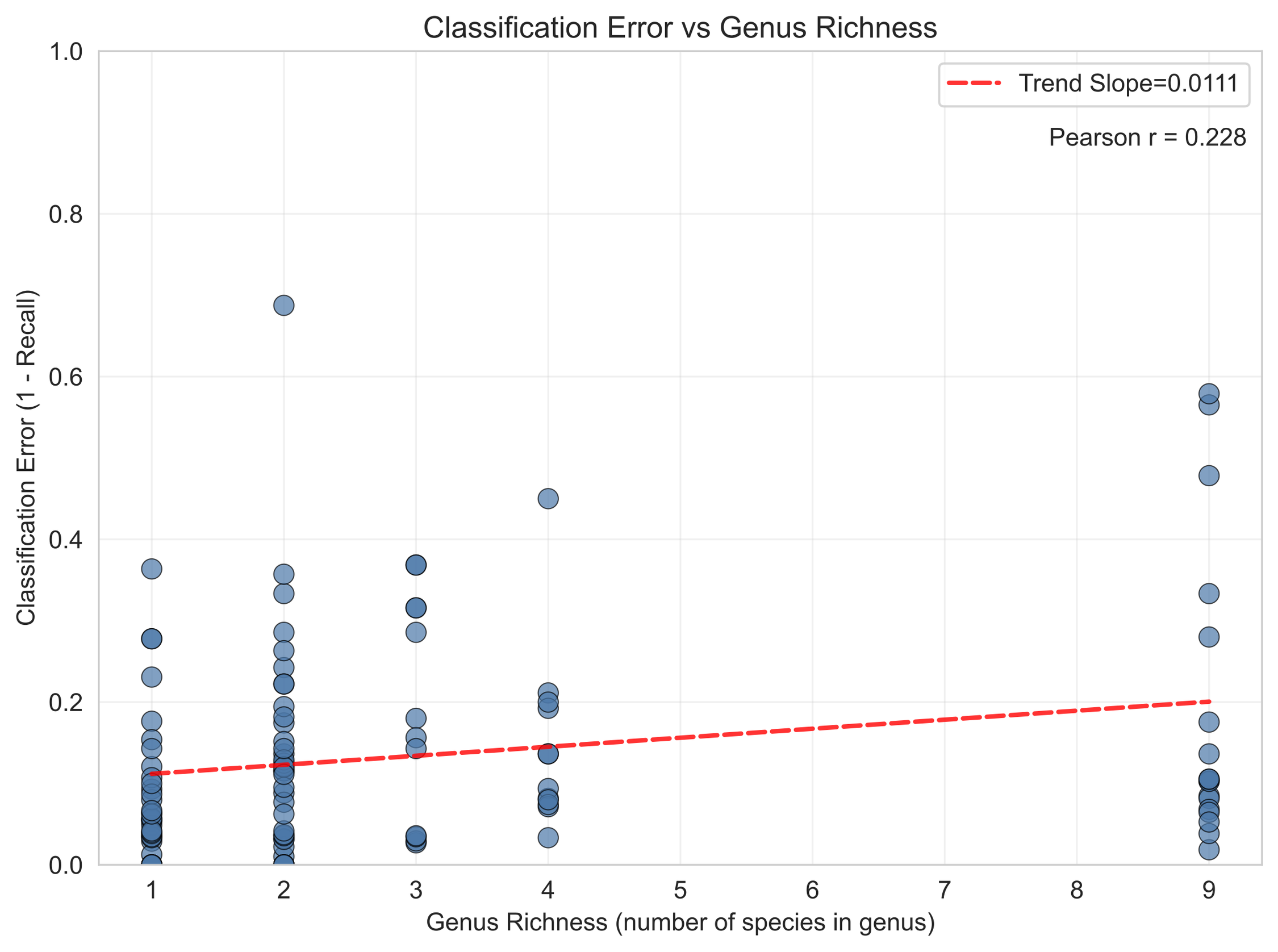

Supplement: S4 Fig — Species-level classification error (1 – recall) plotted against genus richness (number of species within each genus). Each point represents a single species. The dashed red line indicates the linear regression trend (slope = 0.0111), and Pearson’s correlation coefficient is r = 0.228. (TIFF) [file pone.0349646.s004.tiff]
